# Omecamtiv Mecarbil: A Novel Mechanistic and Therapeutic Approach to Chronic Heart Failure Management

**DOI:** 10.7759/cureus.12419

**Published:** 2021-01-01

**Authors:** Pooja H Patel, Michelle Nguyen, Rubi Rodriguez, Salim Surani, George Udeani

**Affiliations:** 1 College of Pharmacy, Texas A&M University, Kingsville, USA; 2 Internal Medicine, Corpus Christi Medical Center, Corpus Christi, USA; 3 Internal Medicine, University of North Texas, Dallas, USA

**Keywords:** heart failure, cardiac contractility, omecamtiv mecarbil

## Abstract

Heart failure (HF) is a major public health problem in the United States as well as worldwide. Chronic heart failure is a syndrome of reduced cardiac output resulting from impaired ventricular function, impaired filling, or a combination of both. Associated symptoms include dyspnea, fatigue, and decreased exercise tolerance. HF has a marked effect on morbidity and mortality, given limited therapeutic choices. The first line of therapeutic agents indicated in heart failure are beta-blockers. Other drugs and therapeutic modalities employed in HF treatment include angiotensin-receptor blockers (ARBs), sacubitril (neprilysin inhibitor) combination with the ARB, valsartan, small doses of aldosterone receptor antagonists (ARAs) in the setting of angiotensin-converting enzyme (ACE) inhibitors, and beta-blockers. Additionally, the sodium-glucose transporter-2 inhibitor, dapagliflozin in the setting of ACE inhibitors, ARBs, or sacubitril-valsartan plus beta-blocker have been employed. Other therapeutic modalities have included loop diuretics, digoxin, the hydralazine-isosorbide dinitrate combination, ivabradine, the inotropes, dobutamine, milrinone, and dopamine. Decreased cardiac contractility is central to the systolic HF. Therapeutic agents employed to increase cardiac contractility in HF are limited because of their mechanistic-related adverse effect profiles. Omecamtiv mecarbil (OM) is a first of its class cardiac myosin activator that increases the cardiac contractility by specifically binding to the catalytic S1 domain of cardiac myosin, to be employed in heart failure treatment. This agent has demonstrated benefit in reducing heart rate, peripheral vascular resistance, mean left arterial pressure, and left ventricular end-diastolic pressure in the animal models. Additionally, OM is known to improve systolic wall thickening, stroke volume (SV), and cardiac output (CO). OM increases systolic ejection time (SET), cardiac myocyte fractional shortening without significant increase of LV dP/dtmax, myocardial oxygen consumption, and myocyte intracellular calcium. The benefits of OM have been demonstrated through key trials, as (i) The Acute Treatment with Omecamtiv mecarbil to Increase Contractility in Acute Heart Failure (ATOMIC-AHF), and (ii) The Chronic Oral Study of Myosin Activation to Increase Contractility in Heart Failure (COSMIC-HF). The Global Approach to Lowering Adverse Cardiac Outcomes Through Improving Contractility in Heart Failure (GALACTIC-HF) trial is ongoing and can help provide further clinical data. OM provides a novel mechanism and therapeutic approach to managing patients with HF. Preclinical and clinical data suggest that OM capability can improve cardiac function, decrease ventricular wall stress, reverse ventricular remodeling, and promote sympathetic withdrawal.

## Introduction and background

Heart failure (HF) is a significant public health challenge, with a prevalence of more than 5.8 million individuals in the United States (US) and greater than 23 million patients worldwide [1]. Acute decompensated HF (ADHF) is the primary cause for hospitalization in the United States among the elderly individuals with age greater than 65 years, and causing almost 1 million hospitalizations annually [2,3]. Prevalence of HF was higher among men in the Framingham Heart study. The prevalence was eight per 1000 for ages between 50 and 59 years, increasing to 66 per 1000 for those between the ages of 80 to 89. The prevalence of heart failure in women was observed to increase from eight cases/1,000 in the 50 to 59 years category to 79 cases/1,000 in those between 80 and 89 years of age [4]. Over the next four decades, the HF in the US is projected to rise. An estimated 772,000 new HF cases are projected to occur by the year 2040 [5]. Current available HF treatments have severe shortcomings and safety issues. There is a significant need for the newer agents that can help improve long-term outcomes and symptomatic relief. HF has a marked effect on morbidity and mortality, with limited therapeutic choices, particularly in the inotropic therapy environment [6].

## Review

Overview of chronic heart failure

In chronic heart failure (CHF), also known as congestive heart failure, there is decreased capacity of the heart to circulate and propel adequate blood and oxygenation throughout the body [5]. CHF is a syndrome, in which there is decreased cardiac output (CO) resulting from poor ventricular ejection, impaired filling, or a combination of both. As a result of impaired cardiac pumping ability, the heart becomes incapable of keeping up with the body’s oxygen demands. This results in symptoms such as dyspnea, fatigue, and decreased exercise tolerance. HF may contribute to fluid retention, pulmonary congestion, and peripheral edema. In some instances, the disorder can be mild, triggering symptoms noticeable only during exercise. In other situations, the condition may be quite severe, triggering life-threatening symptoms [6]. Patients at higher risk of developing HF are typically older, African-American, and male. Hypertension, end organ failure (renal failure), diabetes, hyperlipidemia, and coronary artery disease can increase the risk of heart failure. There is genetic predisposition to heart failure, additionally, patients who smoke, and are obese are also at a higher risk [7].

Based on ejection fraction, heart failure can be categorized into three broad categories. These include: (1) Heart failure with reduced ejection fraction (HFrEF), EF > 40%; (2) heart failure with preserved ejection fraction (HFpEF), EF >50%; and (3) heart failure with mid-range EF (HFmrEF), also referred to as HFpEF-borderline and HFpEF-improved with EF in HFrEF improved > 40%; EF of 40-49% and 41-49% based on US and European guidelines, respectively [7, 8]. The New York Heart Association (NYHA) guideline has been used for almost a century as a foundational tool for HF risk stratification and measure of heart failure severity [9]. Besides this, the clinicians have also used the American College of Cardiology Foundation (ACCF) and American Heart Association (AHA) guidelines [7].

Cardiomyopathy frequently results in heart failure [10]. There are five known cardiomyopathies: dilated, hypertrophic, restrictive, arrhythmogenic, right-ventricular, and unclassified. The various causes of cardiomyopathy include cardiovascular, genetic, infection, inflammation, metabolic, and pregnancy. Myocardial dysfunction is the most frequent pathway to most cardiomyopathies [11]. While histologic findings for these cardiomyopathies are nonspecific, they include cellular necrosis, myocyte hypertrophy, and fibrosis [12]. Most cardiac pathology-associated myocardial dysfunction results in abnormal myocyte growth [13]. Additionally, a cascade of gene activation that stimulates cardiac remodeling ensues. Myocardial cell hypertrophy, cardiac dilatation, increased interstitial matrix formation are trademarks of cardiac remodeling [14].

Drug therapy in heart failure

Current heart failure therapy concepts rely on two mechanisms. The most successful pathway is blockage of neurohormonal activation with ACE inhibitors, beta-blockers, and/or aldosterone receptor blockers [15]. The second mechanism is the increased cardiac contractility. This is achieved indirectly via activation of second-messenger signaling pathways that increase cardiac myocyte intracellular calcium concentration by beta-adrenergic receptor agonists and phosphodiesterase inhibitors. Unfortunately, these agents are associated with increased heart rate and myocardial oxygen demand. This results in significant arrhythmias and hypotension, leading to increased mortality [16].

Direct cardiac sarcomere contractility activation has been demonstrated to improve cardiac performance without adverse effects. Sarcomere consists of thin and thick interdigitating filaments. Myosin is the core of the thick filament and uses chemical energy derived from ATP hydrolysis for contraction. Myosin motors act on the thin filaments, which consist of actin and the regulatory complex, troponin-tropomyosin. Free calcium levels in resting muscles low thus, regulatory proteins prevent myosin from interacting with actin. Calcium is routinely and transiently released from the sarcoplasmic reticulum into the cytoplasm with every heartbeat. Calcium then binds to troponin, allowing myosin to interact with actin filaments for the contraction to occur. As calcium is removed from the cytoplasm, the muscle relaxes. Direct activation of cardiac sarcomere can be conceptually achieved either via sensitizing the regulatory proteins to calcium or activating cardiac myosin directly [17]. This concept was further explored in drug development via high-throughput screening of the cardiac myosin, adenosine triphosphatase (ATPase) was conducted. This process resulted in the discovery of a small-molecule activator of cardiac myosin. This molecule was further enhanced for physical properties, potency, and pharmacokinetics, resulting in the synthesis of omecamtiv mecarbil [18]. This agent was observed to improve cardiac function in the absence of myocardial oxygen consumption changes during preclinical trials in a dog heart failure model [19].

Various markers such as heart rate (HR), blood pressure (BP) are essential for monitoring therapies and disease progression to prevent morbidity in heart failure patients. Other markers used to monitor drug therapy in heart failure include cardiac output (CO) and systemic vascular resistance (SVR). For example, the employment of vasodilators in heart failure increases CO via decreasing SVR, with inotropes deployed for blood pressure support. Mean arterial pressure (MAP) could also be readily monitored in this population, given that MAP = CO SVR. Cardiac index (CI) and brain natriuretic peptide (BNP) have also been used as markers to monitor heart failure therapy. OM is known to improve systolic wall thickening, stroke volume (SV), and CO [19,20].

Beta-blockers

The first line of drugs indicated in heart failure are beta-blockers, which are recommended for use, regardless of NYHA class. Beta-blockers block the SNS activation by blocking epinephrine and norepinephrine and provide benefits in controlling heart rate and reducing the risk of arrhythmias [21]. Individuals with less severe heart failure, including those with left ventricular HFpEF and no symptoms, have demonstrated the most long-term benefit [22].

Angiotensin-converting enzyme inhibitors

Angiotensin-converting enzyme (ACE) inhibitors are recommended for use in all individuals with left ventricular systolic dysfunction or HfrEF, irrespective of functional class, except in those cases of intolerance or a contraindication, such as angioedema exists. Mortality reduction in heart failure observed with these agents has hovered around 30% [23].

Angiotensin-receptor blockers

Angiotensin-receptor blockers (ARBs) are recommended in individuals with intolerable adverse effects from ACE inhibitors (predominantly cough). Decreased mortality in various randomized controlled trials has ranged from 30%-45% [24-27]. ACE inhibitors and ARBs block neurohormonal activation of the renin-angiotensin-aldosterone system (RAAS), resulting in vasodilation and improved ejection fraction [28].

Neprilysin inhibitors and angiotensin-receptor blockers (ARBs)

The employment of sacubitril (neprilysin inhibitor) and use with the ARB, valsartan in HF patients with mild to severe symptoms, NYHA class II-IV has further reduced the risk for cardiovascular death or heart failure associated hospitalization by 20% [29].

Beta-blockers, hydralazine and nitrates

In individuals who cannot tolerate ACE inhibitors or ARBs, the combination of hydralazine and isosorbide dinitrate should be considered; however, the decrease in mortality is not as profound as with ACE inhibitors and ARBs [30]. This benefit has been more profound in African-American patients, with severe symptomatic heart failure (NYHA class III or IV), where hydralazine and isosorbide dinitrate combination in addition to ACE inhibitor or ARB and beta-blocker, has been shown to favorably affect myocardial remodeling as well as mortality [31].

Aldosterone antagonists

The addition of small doses of aldosterone receptor antagonists (ARAs) in the setting of ACE inhibitors and beta-blockers may provide added diuresis, improved symptoms, and ejection fraction. These agents also reduce morbidity and mortality for patients in NYHA Class II-IV [32-34].

Sodium-glucose-transporter-2 inhibitor

The employment of the sodium-glucose transporter-2 inhibitor, dapagliflozin in the setting of ACE inhibitors, ARBs, or sacubitril-valsartan plus a beta-blocker has also been shown to reduce all-cause mortality and worsening HF in adults with NYHA Class II-IV with or without diabetes mellitus [35].

Loop diuretics

Loop diuretics reduce blood volume, which decreases edema and congestion in patients with HF. Most patients will need a loop diuretic for symptom relief. These are the only agents that have demonstrated short-term symptomatic benefits. Additionally, diuretics decrease pulmonary capillary wedge pressure as well as increase exercise capacity [36].

Digoxin

Digoxin has been used in the treatment of HF for decades. It can cause a small increase in cardiac output, improves the patient symptoms, and helps in decreasing HF-related hospitalizations. It is best reserved for individuals with symptomatic NYHA class II to IV heart failure; however, it provides no survival advantage in comparison to placebo [37]. Digoxin has been shown to reduce hospitalizations in heart failure patients, as well as reduce mortality at low serum concentrations [38-40].

Ivabradine

Ivabradine has been demonstrated to decrease hospitalization risk in patients with mild to moderate symptoms, within NYHA classes II-III who are in normal sinus rhythm with a resting heart rate greater than or equal to 70 beats per minute (BPM) [41]. Its ability to decrease the heart rate via selective inhibition of the current in the sinoatrial node has been employed in the treatment of the heart failure [42].

Inotropic agents

The use of intravenous inotropes can be helpful in afterload reduction for hospitalized patients unresponsive to oral agents, with decompensated heart failure with low cardiac output and evidence of end-organ hypoperfusion or hypotension. However, these agents' routine use for inpatient management of acute decompensated HF is limited due to a lack of evidence for a survival benefit and concerns regarding the risk of increased mortality and adverse side effects [43]. Additionally, only select patients who cannot be weaned from intravenous therapy while inpatient may be candidates for chronic outpatient inotropic infusion therapy. This strategy is commonly used in patients who are awaiting mechanical circulatory support or heart transplantation. Common inotropic agents include dobutamine, milrinone, and dopamine.

Several agents described above have been used in the management of heart failure, with some successes. Unfortunately, progress in the development of therapeutic agents for heart failure management has been plodding. Omecamtiv mecarbil is a novel agent, recently granted Fast Track designation by the Food and Drug Administration for the potential treatment of chronic heart failure in HFrEF.

Omecamtiv mecarbil (OM)

Myocardial contractility is at the core of HF progression, compromising cardiac output (CO), initially upon effort, and at rest in later stages of the disease [44]. Omecamtiv mecarbil (OM) is a first in its class cardiac myosin activator that increases cardiac contractility via specifically binding to the catalytic S1 domain of cardiac myosin. Normally, the binding between myosin-adenosine triphosphate (ATP) and actin filaments is weak, until hydrolysis of ATP to adenosine diphosphate (ADP) and inorganic phosphate (Pi) occurs. After that Pi is subsequently released from the myosin. The remaining myosin-ADP complex then remains firmly attached to the actin in a very stable force-generating complex. Its slow dissociation happens once the molecule of ATP binds myosin causing a conformational change. Hence, the hydrolysis of ATP to ADP-Pi and consequently, Pi release from myosin demonstrates a transition from a weakly actin-binding state to a strongly actin-binding state [6]. This transition is a rate-limiting step during the whole actin-myosin ATPase cycle [45]. Through the cardiac myosin activation, OM induces the conformational changes that augment the speed of ATP hydrolysis, which causes the release of Pi and accelerating the transition rate from a weakly bound to a firmly bound force-producing state. Physiologically, only about 10-30% of total cardiac myosin heads interact with actin filaments [46]. OM increases the proportion of myosin heads that are tightly bound to actin and creates a force-producing state that is independent of an increase in intracellular calcium [47]. Ultimately, OM prolongs total systole duration by augmenting the entry rate of myosin into a force-generating state. This increases the formation of active cross-bridges and thus, facilitating a stronger cardiac contraction.

OM has demonstrated benefit in reducing heart rate, peripheral vascular resistance, mean left arterial pressure, and left ventricular end-diastolic pressure in previous experimental canine studies. OM has also been shown to improve the CO, Stroke Volume, (SV) and systolic wall thickening. Besides OM was also shown to increase systolic ejection time (SET) as well as cardiac myocyte fractional shortening without a significant increase in LV dP/dtmax, myocardial oxygen consumption, and myocyte intracellular calcium [19].

The population that may benefit from OM was described in the GALACTIC-HF trial as those with EF of less than or equivalent to 28% [48]. Additionally, OM has demonstrated minimal limitations with typical adverse effects on heart rate, blood pressure, potassium homeostasis, and renal function observed with currently available HF therapies. Thus there may be benefits from OM therapy in that it may not interfere with initiation or up-titration of other essential therapies. Several phases 2 and 3 clinical trials conducted with Omecamtiv mecarbil to date are discussed (Table 1) [48-50].

**Table 1:** Clinical trials

Clinical Trial	Phase	Study Design	Primary and Secondary Outcomes	Results	Status
ATOMIC-AHF [49] "Acute Treatment with omecamtiv mecarbil to Increase Contractility in Acute Heart Failure"	II	Multicenter, prospective, double-blind, randomized, placebo-controlled, dose-escalation, sequential-cohort trial comparing omecamtiv mecarbil versus placebo in individuals hospitalized for acute heart failure. Patients were randomized to receive a double-blind, 48-hour intravenous infusion of placebo or OM in 3 sequential, escalating dose cohorts targeting mean OM plasma concentrations at 48 h of 115 ng/mL, 230 ng/mL, and 310 ng/ml. 613 patients enrolled. OM was administered via a 4-hour loading dose, followed by a 44-hour maintenance dose infusion. The loading doses were as follows for these cohorts: 7.5 mg/hr (Cohort 1), 15 mg/hr (Cohort 2) and 20 mg/hr (Cohort 3). The maintenance infusions were as follows for these Cohorts: 1.5 mg/hr (Cohort 1), 3 mg/hr (Cohort 2), and 4 mg/hr (Cohort 3).	The primary efficacy endpoint evaluated dyspnea improvement without experiencing worsening heart failure or death from any cause by 48 hours. The secondary and exploratory endpoints included evaluations of symptoms and clinical events (e.g., death from any cause or worsening HF within 7 days, change from baseline in NT-proBNP at 48 hours, dyspnea numerical response AUC through day 5, patient global assessment response, the incidence of supraventricular tachycardia (SVT) or ventricular tachycardia (VT) requiring treatment, days alive out of the hospital, through 30 days, length of initial hospital stay, IV loop diuretic use, CHF drugs upon discharge and renal impairment.	In 606 patients who received investigational treatment, OM did not improve the primary endpoint of dyspnea or any pre-specified secondary outcome when compared with placebo. The exploratory post hoc logistic regression analysis across all cohorts however demonstrated greater dyspnea response rates with higher total dose. OM appeared well tolerated, with approximately dose-proportional pharmacokinetic properties associated with prolonged ventricular systole and decreased left ventricular end-systolic dimension.	Completed
COSMIC-HF [50] "Chronic Oral Study of Myosin Activation to Increase Contractility in Heart Failure"	II	A pharmacokinetic, randomized, placebo-controlled study. The purpose of COSMIC-HF trial was to investigate whether a pharmacokinetic-guided dose titration approach oral OM administered over 20 weeks would result in tolerable plasma drug concentrations associated with improved pharmacodynamic outcomes of ventricular systolic function and favorable ventricular remodeling. In this study, 758 patients were screened, and 448 patients with stable, symptomatic heart failure and left ventricular ejection fraction (LVEF) <40% enrolled and randomly assigned to OM treatment regimens. There were 150 patients in the 25 mg twice daily (fixed-dose) group, 149 patients in the 25 mg, titrated to 50 mg twice daily (pharmacokinetic-titration) group, and 149 in the placebo group. The patients received study treatment or placebo for 20 weeks, and followed up at week 24 post-randomization. Patients in the pharmacokinetic-titration group were treated with 25 mg OM twice daily for 2 weeks to achieve steady-state. The dose was titrated up to 50 mg at week 8 if the trough OM concentration at 2 weeks was less than 200 ng/mL. Patients were continued on 25 mg twice daily through the end of the study if the trough OM concentration was 200 ng/mL or higher.	The study's primary endpoint was the maximum concentration of OM at weeks 2 and 12 visits and the trough concentration at weeks 2, 8, 12, 16, and 20 visits. The study's secondary endpoints were changes from baseline in left ventricular end-systolic, and end-diastolic diameters, stroke volume, heart rate, systolic ejection time, and concentration of NT-proBNP in plasma at week 20.	OM dosing guided by pharmacokinetics was observed to achieve plasma concentrations associated with improved cardiac function and decreased ventricular diameter. The prespecified secondary efficacy endpoints in the pharmacokinetic-titration cohort at 20 weeks, all differed significantly from those in the placebo group. Specifically, the left ventricular end-systolic and end-diastolic diameters and heart rate were reduced in the PK group compared within the placebo group at week 20, but not in the fixed-dose group. Concentrations of NT-proBNP in plasma at 20 weeks were reduced in both OM groups. The mean maximum concentration of OM at 12 weeks was 200 (SD 71) ng/mL, and 318 (129) ng/mL for the fixed-dose category and pharmacokinetic-titration group respectively.	Completed
GALACTIC-HF [48] "Global Approach to Lowering Adverse Cardiac Outcomes Through Improving Contractility in Heart Failure"	III	International multicenter, randomized, double-blind, placebo-controlled, event-driven cardiovascular outcomes study. The objective of the investigation is to test the hypotheses that OM safely improves symptoms, prevents clinical HF events, and delays cardiovascular death in individuals with chronic HF. The study thus far has enrolled over 8,000 patients randomized to receive either a placebo or an OM pharmacokinetic-guided dose titration strategy of 25, 37.5, or 50 mg twice daily, administered orally. The GALACTIC-HF is the first trial to address the hypothesis that selectively increasing cardiac contractility with OM in patients with HF results in improved clinical outcomes.	The study's primary outcome is time to cardiovascular death or the first HF event. The secondary outcome is cardiovascular death.	The investigation is expected to conclude by January 2021.	Ongoing

## Conclusions

OM provides a novel mechanism and therapeutic approach to managing patients with HF. Preclinical and clinical data demonstrated capability in improved cardiac function, decreased ventricular wall stress, the reversal of ventricular remodeling, and promotion of sympathetic withdrawal. Prior experimentation attempts at increasing cardiac contractility with oral agents in HF have resulted in an increased risk of mortality at pharmacologically effective doses. Via a novel mechanism of action, OM can increase cardiac contractility without predisposing patients to adverse effects seen with other inotropic agents. Additionally, OM has not demonstrated limitations such as adverse effects on blood pressure, heart rate, potassium homeostasis, or renal function that have typically limited the employment of the current HF therapies. Ongoing and future clinical trials will continue to evaluate OM’s value in HF therapy and its benefit in preventing HF events and CV death.
